# Drug History and SLE Laboratory Findings Among Iraqi Patients: A Hospital‐Based Study

**DOI:** 10.1002/hsr2.70493

**Published:** 2025-03-19

**Authors:** Hashim Talib Hashim, Ahmed Dheyaa Al‐Obaidi, Mustafa Najah Al‐Obaidi, Hossam Tharwat Ali, Idris Sula, Hozifa Alfaki, Abdelrahim Elamin, Alaa Hamza Hermis, Assalah Othman, Ahmed Qasim Mohammed Alhatemi

**Affiliations:** ^1^ College of Medicine Warith Al‐Anbiyaa University Karbala Iraq; ^2^ College of Medicine University of Baghdad Baghdad Iraq; ^3^ Qena Faculty of Medicine South Valley University Qena Egypt; ^4^ College of Applied Sciences Sulaiman Al Rajhi University Bukayriah Saudi Arabia; ^5^ Omdurman Islamic University Omdurman Sudan; ^6^ Nursing Department Al‐Mustaqbal University College Babylon Iraq; ^7^ D'Youville University School of Pharmacy Buffalo New York USA; ^8^ Department of Internal Medicine Al Nasiriyah Teaching Hospital Nasiriyah Iraq

**Keywords:** ANCA, biological therapy, clinical features, drugs, immunotherapy, laboratory results, NSAIDs, RF, SLE, vitamin D3

## Abstract

**Background and Aims:**

Systemic lupus erythematosus (SLE) is a recurrent and remitting autoimmune disease that affects many organ systems. It is more common in women of reproductive age, with a 9:1 female preponderance. Due to the lack of data from developing countries, our study aims to provide comprehensive insights into the drug history and SLE laboratory findings among Iraqi patients.

**Methodology:**

It is a cross‐sectional study at the Baghdad Medical Complex in Iraq. All SLE patients who attended the outpatient clinic between September and December 2022 were included. The patients included those who came for follow‐up, deterioration of their symptoms, or a first‐time diagnosis.

**Results:**

SLE is much more prevalent among female patients. The ratio of female and male patients in our study was 19:1. This disease is more prevalent in the reproductive age group, with the mean age of our patients being 34 years, although the range spans from 15 to 85 years. The most prevalent signs and symptoms among the patients were photosensitivity (79.5%), hair loss (74%), and malar rash (72.5%). Most patients had positive antinuclear antibodies and anti‐double‐stranded DNA antibodies, with 73.5% and 65% testing positive, respectively.

**Conclusion:**

In conclusion, this study represents an important step in exploring SLE within Iraq, providing invaluable insights into its management and laboratory findings. The findings contribute to the global understanding of SLE. This study underscores the significance of Iraq's genetic backgrounds and environmental conditions, which are different from those of other countries, in the process of enhancing the global medical literature and expanding our understanding of SLE patterns around the world.

## 1 Introduction

Systemic lupus erythematosus (SLE) is a recurrent and remitting autoimmune disease that affects many organ systems. It is more common in women of reproductive age, with a 9:1 female preponderance [1]. The interaction of genes with environmental variables causes many immunologic changes, culminating in chronic immune reactions to autologous nucleic acids. Both innate and adaptive immune responses are active in SLE. Autoantibodies or immune complex depositions induce tissue damage in the kidneys, heart, arteries, central nervous system, skin, lungs, muscles, and joints, resulting in considerable morbidity and death [2, 3].

Serological tests, such as the presence of autoantibodies in circulation, may be very helpful to the clinician [3]. Several laboratory abnormalities, including positive serum autoantibodies (such as antinuclear antibodies [ANAs], anti‐double‐stranded [ds] DNA, and anti‐Smith antigen [Sm]) and low complement levels, are indicative of SLE [4]. An ANA test is the admission criterion for the current categorization of SLE, considering its high sensitivity for SLE. Anti‐dsDNA testing is usually done following a positive ANA test since these antibodies are among the most identified autoantibodies associated with SLE [5].

SLE therapy aims to achieve symptom control and remission. Hydroxychloroquine (HCQ) is the first treatment, followed by glucocorticoids and methylprednisolone in cases of end organ damage. Immunosuppressants are usually used when relapses occur. HCQ therapy improves SLE clinical conditions by inhibiting inflammatory signaling pathways, lowering anti‐DNA autoantibodies, and restoring complement activity [6]. The primary and most important benefit of HCQ is its ability to regulate the activity of SLE disease. This means that it can improve current clinical symptoms, reduce levels of serum markers in the blood, lower activity scores, avoid a sudden flare‐up of the disease, and maintain a state of remission when used over a long period of time. Recent research has indicated that HCQ has the potential to inhibit early mediators such as the B cell activating factor (BAFF) and interferon (IFN), hence reducing the levels of IFN‐γ‐induced protein 10 (IP‐10) in individuals with partial or new‐onset SLE [7]. However, it is associated with a risk of retinal toxicity [1]. Steroid constitutes an essential part of SLE management. Increasing the prednisolone dose may improve serological abnormalities, but repeated increases can lead to steroid‐related toxicity. Steroids can cause short‐term and long‐term adverse effects, such as Cushing's syndrome, weight gain, osteoporosis, infections, glaucoma, and cataracts [8, 9].

Immunosuppressant agents include azathioprine and methotrexate. Azathioprine treatment improves clinical symptoms and anti‐dsDNA levels [9]. Azathioprine's primary side effects are hepatotoxicity and bone marrow suppression. MTX improved serological abnormalities, leading to a lower SLEDAI score and steroid‐sparing effect [10]. However, its main side effects are stomatitis, bone marrow suppression, hepatitis, and pneumonitis [4]. Additional treatments were also used, and mycophenolate mofetil (MMF) has demonstrated potential in this field. MMF exerts various effects on the immune system. The one that has been explained the most is its specific inhibition of the purine biosynthesis‐related enzyme inosine monophosphate dehydrogenase [11]. Cyclophosphamide is a widely utilized immunosuppressant in medical practice. It is a nonspecific alkylating drug that primarily targets cells undergoing proliferation. Cyclophosphamide suppresses the growth of T and B lymphocytes and impairs the reaction of lymphoblasts to antigen stimulation in individuals. It is often regarded as the primary option for treating lupus nephritis, particularly in cases of severe lupus nephritis [12]. There are several biologic therapies that have been utilized to manage lupus, including anti‐BLyS (belimumab), which targets B‐lymphocyte stimulator to reduce the survival of B cells; Rituximab, an anti‐CD20 monoclonal antibody that depletes B cells; and anti‐IFN therapies, which inhibit the type I IFN pathway implicated in the pathogenesis of lupus [13, 14, 15, 16]. On the other hand, NSAIDs are used to treat serositis and musculoskeletal complaints [1]. They can cause some toxic effects, which require caution and follow‐up. Moreover, correcting vitamin D deficiency improves SLE disease activity, and it has appeared to neutralize antichromatin and anti‐dsDNA antibodies [17, 18].

Due to the lack of data from developing countries, our study aims to provide comprehensive insights into the drug history and laboratory findings associated with SLE among Iraqi patients, thereby contributing valuable information to the global understanding of the disease.

## 2 Methodology

### 2.1 Study Design, Population, and Recruitment Procedure

We conducted a cross‐sectional study at the Baghdad Medical Complex in Iraq. All SLE patients who attended the outpatient clinic between September and December, 2022, were included. The patients included those who came for follow‐up, deterioration of their symptoms, or a first‐time diagnosis. Patients who refused to participate or those with incomplete data were excluded. Data collection was performed based on a convenience sampling type, using the patients' medical records and medical histories.

### 2.2 Study Tool

The questionnaire was divided into four domains. Demographic data domain included age, gender, BMI, marital status, smoking status, highest education level, COVID‐19 vaccination status, family history of SLE, time since symptoms first appeared, and time since diagnosis was first made. The second domain included the common signs and symptoms of SLE. The third domain constituted the medical and drug histories of the participants. The last domain consisted of the laboratory tests and immunological markers of the participants.

### 2.3 Validation and Pilot Study

Before the beginning of data collection, the questionnaire was validated and tested for reliability. To validate the content of the survey, five experts in the immunology and rheumatology fields were invited to assess the clarity, comprehension, and relevance of each question to the intended outcome. Post‐validation, a pilot study was conducted on 50 patients with known SLE. The reliability and internal consistency of the survey were assessed using Cronbach's *α*, which revealed good consistency among the questions (*α* = 0.83).

### 2.4 Ethical Considerations

The study was conducted according to the principles expressed in the Declaration of Helsinki. Participation in this survey was voluntary, and patients consented to participate in the study before the survey was conducted. Patients' anonymity and confidentiality were ensured throughout the study, including data collection and analysis. Ethical approval was obtained from the ethics committee at the University of Baghdad, College of Medicine, in August, 2022, with the code 009345.

### 2.5 Statistical Analysis

The data collected through the questionnaire was transferred to Excel format, and the raw data was encrypted in the Excel sheet to perform a better and more precise statistical analysis on the statistics program. The program used for statistical analysis was SPSS version 25. Categorical baseline variables were denoted as frequencies and percentages.

## 3 Results

The study included 200 patients with SLE who were admitted to Baghdad Medical City during August–December, 2022. Their ages ranged from 15 years to 85 years with a mean (±SD) of 34.78 (±9.718). Their BMIs range from 16.90 kg/m^2^ to 42.44 kg/m^2^ with a mean (±SD) of 28.6143 (±4.72209). Table 1 demonstrates the demographic data for the patients.

**Table 1:** The demographic data for the patients.

		Count	%
Gender	Female	190	95.0%
	Male	10	5.0%
Smoking	Active smoker	25	12.5%
	No	111	55.5%
	Passive smoker	64	32.0%
Highest education level	College	66	33.0%
	Illiterate	22	11.0%
	Primary school	39	19.5%
	Secondary school	73	36.5%
Marital status	No	37	18.5%
	Yes	163	81.5%
COVID‐19 vaccination	No	123	61.5%
	Yes	77	38.5%
Family history of SLE	No	136	68.0%
	Yes	64	32.0%
Time since the symptoms first started	3 months to 1 year	51	25.5%
	< 3 months	26	13.0%
	> 1 year	123	61.5%
Time since the diagnosis was first made	3 months to 1 year	46	23.0%
	< 3 months	40	20.0%
	> 1 year	114	57.0%

In Table 2, the distribution of signs and symptoms among the patients is evaluated and presented. The most prevalent signs and symptoms among the patients were photosensitivity (79.5%), hair loss (74%), and malar rash (72.5%).

**Table 2:** The prevalence of signs and symptoms among the patients.

Signs and symptoms	Count (%)
Malar rash	145 (72.5%)
Discoid rash	59 (29.5%)
Mucosal ulcers	129 (64.5%)
Photosensitivity	159 (79.5%)
Arthritis	114 (57.0%)
Any renal disease	71 (35.5%)
Vasculitis	53 (26.5%)
Neurological symptoms	55 (27.5%)
Hair loss	148 (74.0%)
Serositis (pleuritis, pericarditis)	48 (24.0%)
Visual disturbance	79 (39.5%)
Fever	106 (53.0%)
Headache	100 (50.0%)
Hematological disorder	119 (59.5%)

Table 3 shows the drug history among the participants. The study findings also showed that 18% of participants had other connective tissue diseases, while 24.5% had symptoms indicative of inflammatory arthritis. Additionally, 22.5% of the participants had diabetes, and 33.5% had hypertension. The immunosuppressive agents used by the participants included cyclophosphamide, azathioprine, MMF, cyclosporine, and tacrolimus. The biologic agents used by the participants included belimumab and rituximab. The corticosteroids used by the participants included prednisolone and methylprednisolone. The NSAIDs used by the participants included ibuprofen, naproxen, indomethacin, and celecoxib.

**Table 3:** The drug history of the patients.

Drugs	Count (%)
NSAIDs	98 (49.0%)
Corticosteroids	155 (77.5%)
Immunosuppressants	181 (90.5%)
Biologic therapy	45 (22.5%)
Vitamin D3	116 (58.0%)
Other meds	90 (45.0%)

Table 4 shows the positive and abnormal laboratory findings among the participants. Most patients had positive ANAs and anti‐dsDNA antibodies, with 73.5% and 65% testing positive, respectively.

**Table 4:** Positive and abnormal laboratory findings in patients.

Laboratory findings	Count (%)
ANA	147 (73.5%)
Anti‐dsDNA	130 (65.0%)
Low C3	86 (43.0%)
Low C4	65 (32.5%)
Anti‐La	60 (30.0%)
Anti‐Ro	61 (30.5%)
Anti‐cardiolipin antibodies	43 (21.5%)
Lupus anticoagulant	42 (21.0%)
Rheumatoid factor	61 (30.5%)
Abnormal liver function tests	37 (18.5%)
Abnormal renal function tests	72 (36.0%)
Pyuria	44 (22.0%)
Hematuria	80 (40.0%)
Proteinuria	89 (44.5%)
Cellular casts	18 (9.0%)

## 4 Discussion

SLE presents with a wide range of clinical manifestations. It also varies substantially between different sexes and age groups and is distributed unequally among geographical regions; specifically, SLE occurs more frequently in high‐income countries. A study by Tian et al., which presents a comprehensive systematic analysis and modeling of 112 studies, reveals a significant gap in epidemiological data on SLE for 79.8% of countries worldwide. This finding highlights the need for more comprehensive data collection and research on SLE, especially in areas where there is currently a lack of knowledge. In this context, this study greatly contributes to the global understanding of SLE, an area historically lacking substantial data from Iraq in scientific and healthcare research. The lack of data creates significant barriers to comprehending and resolving SLE‐related issues in the region. The limited availability of Iraqi data can be attributed to various factors, including political instability, conflict, and resource shortages. Nevertheless, Iraq's diverse genetics, environmental conditions, and socioeconomic factors offer valuable contributions to the medical literature, enriching our understanding of SLE trends worldwide [19, 20, 21, 22, 23].

Female patients experience a significantly higher prevalence of SLE. Our study included a higher‐than‐usual ratio of female to male patients (19:1). The male‐to‐female ratio has been found to vary between 1 and 15 in other studies [23, 24, 25, 26, 27, 28]. The condition is more prevalent in women of childbearing age, and although our patients' ages ranged from 15 to 85, the mean age was 34 [26, 29].

Our study demonstrated a significant prevalence of steroid use, with 77.5% of participants reporting usage. This finding aligns closely with the study by Valladales‐Restrepo et al., which reported a similar steroid use rate of 76.1% [30]. However, it contrasts notably with the study by Magro et al., which found a considerably lower prevalence of 45.8% [31]. The discrepancy with Magro et al.'s findings may be attributed to differences in study populations, methodologies, or geographic variations. In terms of nonsteroidal anti‐inflammatory drugs (NSAIDs), our study found that 49.0% of participants used these medications. This rate is significantly higher than the 35.5% reported in the study by Chen et al. [32]. Our study also showed that 58% of patients were taking vitamin D, a finding that closely aligns with the study by Magro et al., which reported a similar rate of 57% [31].

Our study found ANA in 73.5% and anti‐dsDNA in 65% of cases. In comparison, Kumar et al. in India reported ANA at 100% and anti‐dsDNA at 84%; Copper et al. in the United States found ANA at 94% and anti‐dsDNA at 43%; Emorinken et al. in Nigeria showed ANA at 100% and anti‐dsDNA at 69.2%; and Cervera et al. in Europe reported ANA at 96% and anti‐dsDNA at 78% [33, 34, 35, 36]. The variation in ANA and anti‐dsDNA positivity rates across different studies and regions highlights the potential influence of genetic, environmental, and methodological factors. Our study showed that 43% of patients had low C3 and 32.5% had low C4, while the study by Kumar et al. in India reported a low complement level of 30% and a study by Díaz‐Coronado et al. in Colombia found a low complement level of 60.2% [33, 37].

## 5 Limitations

A notable limitation of our study is the relatively small sample size, comprising only 200 patients. While this sample provided valuable insights, its size may limit the generalizability of our findings to the broader Iraqi population with SLE. Additionally, our study was conducted exclusively at Baghdad Medical Complex in Iraq, which may introduce bias as the patient population from this single hospital may not fully reflect the diversity of SLE cases across the country. Furthermore, data regarding the race of the population was not gathered. Therefore, caution is advised when interpreting and extrapolating the results to the wider population of Iraqi patients with SLE.

## 6 Conclusion

In conclusion, this study represents an important step in exploring SLE within Iraq, providing invaluable insights into its management and laboratory findings. The findings contribute to the global understanding of SLE. This study underscores the significance of Iraq's genetic backgrounds and environmental conditions, which are different from those of other countries, in the process of enhancing the global medical literature and expanding our understanding of SLE patterns around the world.

## 7 Recommendations

For future studies, it is recommended to expand sample sizes to enhance the representativeness of findings across diverse populations. One way to decrease the impact of any biases in single‐site studies is to spread out the research across several healthcare institutions or areas. This multicenter approach also enhances the generalizability of the findings by including a more diverse patient population and ensuring that results are applicable to a broader demographic. Moreover, there is a need for more studies not only from Iraq but also from other developing countries to broaden our understanding of health conditions and ensure more inclusive and equitable healthcare practices globally.

## Author Contributions

**Hashim Talib Hashim:** conceptualization, supervision, resources. **Ahmed Dheyaa Al‐Obaidi:** data curation, funding acquisition, formal analysis. **Mustafa Najah Al‐Obaidi:** visualization, investigation, software. **Hossam Tharwat Ali:** methodology, investigation, project administration. **Idris Sula:** data curation, project administration. **Hozifa Alfaki:** validation, writing – original draft, methodology. **Abdelrahim Elamin:** data curation, resources, project administration. **Alaa Hamza Hermis:** validation, methodology, resources. **Assalah Othman:** writing–original draft, writing – review and editing. **Ahmed Qasim Mohammed Alhatemi:** conceptualization, methodology, writing – original draft, writing – review and editing.

## Consent

Informed consent was obtained from the patients to share their data for scientific purposes.

## Conflicts of Interest

The authors declare no conflicts of interest.

## Data Availability

The data that support the findings of this study are available from the corresponding author upon reasonable request.
